# The reciprocity of spatial–numerical associations of vocal response codes depends on stimulus mode

**DOI:** 10.3758/s13421-023-01511-6

**Published:** 2024-01-25

**Authors:** Melanie Richter, Peter Wühr

**Affiliations:** Department of Psychology, TU Dortmund University, Emil-Figge-Strasse 50, 44227 Dortmund, Germany

**Keywords:** SNARC, Number, Location, Reciprocity, Vocal responses

## Abstract

Individuals make faster left responses to small/er numbers and faster right responses to large/r numbers than vice versa. This “spatial–numerical association of response codes” (SNARC) effect represents evidence for an overlap between the cognitive representations of number and space. Theories of the SNARC effect differ in whether they predict bidirectional S-R associations between number and space or not. We investigated the reciprocity of S-R priming effects between number and location in three experiments with vocal responses. In Experiments 1 and 2, participants completed a number–location task, with digits as stimuli and location words as responses, and a location–number task, with physical locations as stimuli and number words as responses. In addition, we varied the S-R mapping in each task. Results revealed a strong SNARC effect in the number–location task, but no reciprocal SNARC effect in the location–number task. In Experiment 3, we replaced physical location stimuli with location words and digit stimuli with number words. Results revealed a regular and a reciprocal SNARC effect of similar size. Reciprocal SNARC effects thus seem to emerge with verbal location stimuli and vocal responses, but not with physical location stimuli and vocal responses. The S-R associations underlying the SNARC effect with vocal responses thus appear bidirectional and symmetrical for some combinations of stimulus and response sets, but not for others. This has implications for theoretical accounts of the SNARC effect which need to explain how stimulus mode affects the emergence of reciprocal but not regular SNARC effects.

## Introduction

A few decades ago, Dehaene and colleagues (Dehaene et al., 1990) made the observation that a left keypress response (with the left hand) is faster and more accurate to smaller numbers compared with larger numbers, whereas a right keypress response (with the right hand) is faster and more accurate to larger numbers compared with smaller numbers. Numerical size can thus influence the selection and execution of spatial responses. This phenomenon, which has been termed the *spatial–numerical association of response codes (SNARC)* effect, implies the existence of associations, or even overlap, between the cognitive representations of number and space, the so-called *spatial–numerical associations*. Since the observation by Dehaene and colleagues (Dehaene et al., 1990), much effort has been dedicated to the investigation of spatial–numerical associations, originally focusing on the SNARC effect and its determinants before expanding to the study of other forms of spatial–numerical associations—for example, in line bisection or random number generation tasks. Research thereby testified to the ubiquity of spatial–numerical associations (Fischer & Shaki, 2014; Gevers & Lammertyn, 2005).

The studies investigating the SNARC effect as prominent evidence of spatial–numerical associations were able to determine several characteristics of the effect. First, the effect also emerges with number as an irrelevant stimulus feature, implying that numerical size is automatically processed even if it is not relevant for task completion (e.g., Dehaene et al., 1993; Gevers et al., 2006; Mapelli et al., 2003). Second, the SNARC effect is context-dependent: The number 5 is, for instance, associated with a right response in a number set ranging from 1 to 5, whereas it is associated with a left response in a number set ranging from 5 to 9 (Dehaene et al., 1993; Fias, 1996). Ben Nathan et al. (2009) even demonstrated that “the effect is instantaneous: What matters is the relative magnitude that a number happens to carry at any given moment” (p. 582). Third, the SNARC effect can also be observed with various stimulus and response sets. If numerical size is visually presented in the form of numbers, number words, or die-faces (number of dots, i.e., numerosity), or even auditorily presented, SNARC effects of similar sizes occur (Nuerk et al., 2005). SNARC effects also emerge with different response modes such as unimanual (e.g., Wood et al., 2008) or vocal responses (e.g., Gevers et al., 2010). The independence of the SNARC effect from stimulus and response modes implies that instead of relying on direct connections between specific stimulus and response codes, the associations between number and space rather seem to rest upon an intervening representational level (e.g., Hubbard et al., 2005; Nuerk et al., 2005).

With their observation that the SNARC effect also arises with vocal responses, Gevers et al. (2010) challenged the prevalent view that *visuospatial coding*—that is, the “correspondence between the position of a number on a continuous left-to-right-oriented representational medium (the mental number line) and the spatial position of the response”—is responsible for the effect (Gevers et al., 2010, p. 181). Instead, the authors were able to show that the SNARC effect at least partly relies on a verbal coding of space in the form of the categorical labels “left” and “right”, which they termed *verbal-spatial coding*. Moreover, Gevers and colleagues (Gevers et al., 2010) demonstrated that a pure verbal encoding of spatial information is sufficient to induce the SNARC effect, emphasizing the role of verbal labeling when spatial locations are encoded and mentally represented.

When investigating the SNARC effect, the question of reciprocity arises. Are the associations between numerical size and spatial location, which underlie the SNARC effect, bidirectional in such a way that spatial stimuli can also activate numerical responses similar to how numerical stimuli can activate spatial responses? Or are they unidirectional in such a way that numerical stimuli can activate spatial responses but spatial stimuli cannot activate numerical responses? So far, some studies have investigated the influence of spatial information onto the processing of numbers but have merely focused on the bidirectionality of spatial–numerical associations in general—that is, without the involvement of response codes. For example, Stoianov et al. (2008) observed that spatial primes can affect performance in a numerical judgement task with “neutral” vocal responses, showing that spatial information affects numerical processing on a central processing stage (i.e., the mental number line; see also Kramer et al., 2011).

Other studies have addressed reciprocal effects between spatial and numerical processing by employing *random number generation* tasks. Loetscher and colleagues (Loetscher et al., 2008), for instance, observed that when attempting to generate random numbers while turning one’s head alternately to the left and right, participants were more likely to produce smaller numbers during leftward movements but larger numbers during rightward movements. Similarly, Loetscher and colleagues (Loetscher et al., 2010) reported that participants were more likely to produce smaller numbers when moving their eyes to the bottom-left, but more likely to produce larger numbers when moving their eyes to the upper-right. These correlational results were extended by Shaki and Fischer (2014), who demonstrated an effect of spatial information processing onto numerical information processing by showing that participants generated smaller numbers before they made a prescribed leftward turn compared with a rightward turn. Nevertheless, it remains unclear if the effect occurred due to the given instruction of the prescribed turn in the form of a stimulus–response (S-R) priming effect, or due to the planning of the spatial action in the form of a response–response (R-R) priming effect. Even though the bidirectionality of spatial–numerical associations could thus be demonstrated, the bidirectionality of true S-R priming effects of spatial-numerical associations such as the SNARC effect have rarely been addressed so far.

Together with a previous study (Richter & Wühr, 2023), this paper tries to close this gap by investigating the reciprocity of the SNARC effect. In doing that, we are the first to directly compare the regular^1^ SNARC effect in a number–location task, which employs numerical stimuli (e.g., 1 and 2) and spatial (i.e., left and right) responses, to a “reciprocal”^2^ SNARC effect in a location–number task, which employs spatial (i.e., left and right) stimuli and numerical responses (e.g., 1 and 2). The regular SNARC effect describes the phenomenon that numerical stimuli influence the selection and execution of spatial responses in such a manner that small numbers prime left responses and large numbers prime right responses. If the SNARC effect is bidirectional, spatial stimuli should also influence the selection and execution of numerical responses in the form of a reciprocal SNARC effect. So far, several theories that have been proposed to account for SNARC effects differ in whether they assume bi- or unidirectional associations between number and space and thus in whether they predict reciprocal SNARC effects or not.

The *mental number line (MNL)* was the first theory proposed in order to account for the SNARC effect (e.g., Dehaene, 2003; Dehaene et al., 1993; Fischer & Shaki, 2014). The MNL assumes that numbers are mentally represented from left to right in an ascending order with small numbers located on the left and large numbers located on the right. When processing numerical sizes, the number activated simultaneously activates the corresponding position on the MNL. The MNL is assumed to be situated between stimulus processing and response selection and to be part of long-term memory (e.g., Dehaene et al., 1993; Ginsburg & Gevers, 2015; Huber et al., 2016; Umiltà et al., 2010). That means, a numerical stimulus activates a number code together with its spatial position on the MNL, which then primes a corresponding spatial response (Dehaene et al., 1993; Fischer et al., 2003; Restle, 1970). Note, however, that the representational overlap of number and space on the MNL does not necessarily imply reciprocal, or even symmetrical, SNARC effects. Rather, it is possible that asymmetries arise either between different stimulus codes and the MNL, or between the MNL and different response codes. For example, it is possible that numerical and spatial stimuli activate the MNL to different degrees.

The *polarity correspondence principle* postulated by Proctor and colleagues (e.g., Proctor & Cho, 2006) constitutes a second account of the SNARC effect. According to this principle, in many binary classification tasks positive polarity is assigned to one stimulus and response alternative, whereas negative polarity is assigned to the opposite stimulus and response alternative. In a typical SNARC task, the stimuli vary on the bipolar dimension of numerical size (small–large) while the responses vary on the bipolar dimension of spatial location (left–right). The polarity correspondence principle assumes that negative polarity is assigned to the categories “small” and “left”, whereas positive polarity is assigned to the categories “large” and “right” (Proctor & Cho, 2006). In case the polarities of stimuli and responses match, faster and more accurate responses are made compared with when they do not match (see Lakens, 2012; Proctor & Cho, 2006; Proctor & Xiong, 2015). In contrast to the MNL, the polarity correspondence principle implies that the associations between number and space are part of working memory (WM) as stimulus and response alternatives are given positive and negative polarities in relation to the opposing alternatives and thus depend on the given stimulus and response set (see Ben Nathan et al., 2009; Gevers et al., 2006; Wühr & Richter, 2022).

In our view, the polarity correspondence principle should predict bidirectional SNARC effects because both numbers and locations are assigned polarities regardless of whether they occur as stimulus or response features. In particular, if participants code “left” and “right” responses as negative and positive polarity, respectively, then “left” and “right” should also be coded as negative and positive, respectively, when they refer to alternative stimuli. The latter assumption is supported by findings demonstrating superior processing of right locations as compared with left locations, a finding that has been attributed to different polarities (e.g., Just & Carpenter, 1975; Olson & Laxar, 1973). Similarly, if participants code “small” and “large” stimuli as negative and positive polarity, respectively, then “small” and “large” should also be coded as negative and positive, respectively, when they refer to alternative responses.

A third account which emphasizes the role of short-term associations between number and space as underlying the SNARC effect is the *working memory (WM) account* by van Dijck and colleagues (e.g., Abrahamse et al., 2016; van Dijck & Fias, 2011; van Dijck et al., 2015). According to this account, the crucial variable is the serial order in which the elements of a stimulus set are stored in WM, with earlier serial positions being associated with left spatial locations, and later serial positions being associated with right spatial locations. The WM account of the SNARC effect has later been extended to a more general theory of serial-order coding in WM, called the *mental whiteboard hypothesis* (Abrahamse et al., 2014, 2017). According to this hypothesis, coding the serial order of items in (verbal) WM is achieved by connecting the items to spatial position markers. These spatial position markers are conceived as “coordinates within an internal, spatially defined system” (Abrahamse et al., 2014, p. 2). Moreover, it is assumed “that [the spatial coding of serial order] spontaneously occurs from left to right” (Abrahamse et al., 2014, p. 2), which provides an account for the SNARC effect with number stimuli and spatial responses (e.g., Abrahamse et al., 2014). In particular, the authors assume that participants spontaneously store the stimulus numbers of a typical SNARC experiment in ascending order, which implies that smaller numbers are tagged to left-side position markers and larger numbers are tagged to right-side position markers, and the position markers subsequently affect the selection of a (congruent or incongruent) spatial response to a stimulus (Abrahamse et al., 2014, 2016). In contrast to the MNL, which assumes that spatial–numerical associations are stored in long-term memory (Dehaene et al., 1993; Ginsburg & Gevers, 2015; Huber et al., 2016), the mental whiteboard hypothesis assumes that they are stored in WM. Moreover, while the MNL accounts for spatial–numerical associations only, the mental whiteboard hypothesis accounts for associations between any ordinal quantity and space (Abrahamse et al., 2014).

Importantly, proponents of the mental whiteboard hypothesis assume a bidirectional relationship between spatial processing and (verbal) serial order memory (e.g., De Belder et al., 2015). Accordingly, retrieval from serial order memory should not only modulate spatial processing, as reflected in the regular SNARC effect, but spatial processing should also modulate retrieval from serial order memory. The authors provided evidence for this assumption by showing that the location of (irrelevant) spatial cues affected the retrieval of letters depending on the serial position of the letter within a to-be-stored list. In particular, left-side cues facilitated retrieval of letters at early list positions, whereas right-side cues facilitated retrieval of letters at later list positions (De Belder et al., 2015). Despite assuming a bidirectional relation between spatial processing and (verbal) serial order memory, it is not immediately clear whether the WM account, or the mental whiteboard hypothesis, would predict a reciprocal SNARC effect. Assume a task in which participants respond to a left or right location stimulus by pressing a key once or twice, and the S-R mapping is varied. Even if participants spontaneously store the stimuli in a canonical order (i.e., from left to right), and spatial tags would be used for coding serial position, it is not clear why these spatial tags should differently prime the one- or two-keypress responses. Moreover, the mental whiteboard hypothesis explicitly assumes spatial position markers for coding serial positions in *verbal* WM. Verbal WM is clearly implicated in the typical SNARC task with alphanumeric stimuli, but not in the reciprocal SNARC task with spatial stimuli. In fact, it is unclear whether spatial position markers are also involved in representing serial order of spatial stimuli (e.g., De Belder et al., 2015; Ginsburg et al., 2017).

In a previous study (Richter & Wühr, 2023), we have already begun to investigate whether SNARC effects are reciprocal or not. More specifically, in two experiments, we investigated whether the processing of spatial stimuli can influence the selection and execution of numerical responses, which would lead to the occurrence of a reciprocal SNARC effect. To do so, we compared the compatibility effect of a number–location task, resembling the typical SNARC task, with the compatibility effect of a location–number task, resembling a reciprocal SNARC task. In the number–location task, one and two dots (Exp. 1) or the digits 1 and 2 (Exp. 2) served as stimuli to which participants responded by pressing a left or right key with two fingers of their dominant hand. In the location–number task, a black square presented in a left/right location served as a stimulus to which participants responded by pressing a key once or twice with the index finger of their dominant hand. Participants completed both tasks twice, once according to a compatible mapping (one–left, two–right; left–one, right–two) and once according to an incompatible mapping (one–right, two–left; left–two, right–one).

As expected, we found a regular SNARC effect in the number–location task of both experiments: Participants responded faster and more accurately in the compatible compared with the incompatible mapping. However, we did not find a reciprocal SNARC effect: In the location–number task, participants’ performance was similar across both mapping conditions. Those results suggest that numerical stimuli can influence spatial responses whereas spatial stimuli cannot influence numerical responses. However, excluding outlier data affected the pattern of results in Experiment 2, where we used digit stimuli: Including outliers in the analysis led to a small, but significant, reciprocal SNARC effect. While reciprocal SNARC effects seemed absent for the majority of participants, small effects emerged in a subsample which showed extreme reaction times and/or error percentages. Thus, the associations between number and space, which underlie the SNARC effect, seem to be at least strongly asymmetrical.

The major aim of the present study was to further investigate the reciprocity of the SNARC effect in three experiments with vocal responses, and to replicate and extend the results of our previous experiments (Richter & Wühr, 2023). In particular, the present experiments differed in three important aspects from the previous experiments. Firstly, we changed the response mode. In the experiments reported in this paper, participants responded vocally, instead of manually, by saying a location word (“left”^3^ or “right”) in the number–location task, and by saying a number word in the location–number task (“one” or “two” in Experiment 1, “one” or “nine” in Experiments 2 and 3). Crucially, Gevers et al. (2010) have shown that SNARC effects of similar size can be obtained with manual as well as with vocal responses. Moreover, Gevers and colleagues concluded that “verbal–spatial coding was the dominant factor in driving the SNARC effect” (Gevers et al., 2010, p. 187) in their experiments, emphasizing the role of verbal rather than visual encoding of spatial information which is then associated with numerical size. We therefore predicted that the results with vocal responses should be similar to the results we obtained with manual responses in the previous study (Richter & Wühr, 2023).

Secondly, in our previous experiments with manual keypress responses, we used the numerical values 1 and 2 both as stimulus and as (manual) response values. To foster a comparison between the results, we used the same stimulus and response values in the present Experiment 1. However, we found it important to investigate the reciprocity of S-R compatibility effects between number and space with different stimulus and response sets to demonstrate the robustness of our results. We therefore used different numerical values (i.e., 1 and 9) in Experiments 2 and 3. A similar increase in the number of sequential keypress responses is less practical for obvious reasons, and would introduce other problems such as differences in the complexity of response alternatives (e.g., Henry & Rogers, 1960; Sternberg et al., 1978).

Thirdly, while we used visuospatial stimuli (left and right physical locations) in the location–number task of our previous experiments, we wanted to investigate whether a consistent use of alphanumeric stimuli would affect the pattern of results. In Experiments 1 and 2 of our present paper, we merely changed the response mode by using alphanumeric responses (“left”/“right” in the number–location task; “one”/“two” or “nine” in the location–number task). In Experiment 3, we also changed the stimulus presentation by using alphanumeric stimuli in the form of number words (“one”/“nine” in the number–location task) and location words (“left”/“right” in the location–number task). Note that while responses in all three experiments were given vocally, stimuli in all three experiments were presented visually in the form of digits or number words in the number–location task and in the form of physical locations or location words in the location–number task. Therefore, we differentiate between vocal responses (in the sense of spoken) and verbal stimuli (in the sense of written).

## Experiment 1

The purpose of Experiment 1 was to investigate the reciprocity of the SNARC effect with vocal responses. Therefore, we compared the compatibility effect of a number–location task (SNARC task) with the compatibility effect of a location–number task (reciprocal SNARC task). In the number–location task, participants responded to the digit 1 or 2 with the vocal response “left” or “right”. Conversely, in the location–number task, participants responded to a left or right stimulus location with the vocal response “one” or “two”. Participants completed both tasks twice, once according to a compatible mapping (1–left, 2–right; left–1, right–2) and once according to an incompatible mapping (1–right, 2–left; left–2, right–1). The compatibility effect served as a measure for the strength of the associations between stimuli and responses which could form three possible patterns. Significant compatibility effects of equal size would suggest bidirectional and symmetrical associations. Significant compatibility effects of different sizes would suggest bidirectional but asymmetrical associations. A significant compatibility effect in the typical number–location task and a nonsignificant compatibility effect in the reciprocal location–number task would, contrarily, indicate unidirectional associations. Note that the terms reciprocity and uni-/bidirectionality thus refer to the direction of effects, whereas the terms symmetry and asymmetry refer to the magnitude of effects.

### Methods

#### Participants

Similar SNARC effects can be obtained with manual as well as with vocal responses in a typical SNARC task, that is, employing numerical stimuli and spatial responses. Gevers et al. (2010, Experiment 1), for example, reported a vocal SNARC effect of η_p_^2^ = .33, which was not statistically different from a SNARC effect with manual responses. Since we were interested in a so-far unknown reciprocal SNARC effect with vocal responses, which might have smaller effect sizes, we decided to reduce the effect size estimate and use an effect size of η_p_^2^ = 0.2 for a power analysis. We conducted the power analysis with the software MorePower (Campbell & Thompson, 2012) revealing that a sample size of 54 participants would be required to detect an effect of this size with high power (1 − β = .95) at the standard alpha-error probability of .05.

Sixty students (53 female, seven male) with a mean age of 20.633 years (*SD* = 3.551) voluntarily participated in our experiment. According to self-report, 57 participants were right-handed, whereas three participants were left-handed. All participants reported normal (*N* = 30) or corrected-to-normal vision (*N* = 30). Prior to participation, all volunteers gave their informed consent. They were compensated by receiving either course credit or a payment of 10 Euro in exchange for participation. The local Ethics Committee at TU Dortmund University approved the experimental protocol for our study (GEKTUDO_2022_36).

#### Apparatus and stimuli

Participants sat in front of a 19-inch color monitor with a viewing distance of approximately 50 cm. The software E-Prime 3.0 (Psychology Software Tools; Sharpsburg, PA, USA) controlled the presentation of stimuli and registered responses (i.e., vocal responses, reaction times). A small plus sign (Courier font, size 18 pt) served as a fixation point and was presented at the screen center at the beginning of each trial. All stimuli were presented in black on a white background. In the number–location task, the Arabic digits 1 and 2 (Times New Roman, size 40 pt) served as stimuli and were presented at screen center. Participants responded vocally to the stimuli by saying “left” or “right” into a microphone, which was aligned to the participants’ midline and stood directly in front of them. The microphone was connected to the voice-key of the Chronos console (Psychology Software Tools; Sharpsburg, PA, USA), which registered reaction times and recorded the participants’ vocal responses. Each vocal response was stored in a separate audio-file and later checked in terms of accuracy. In the location–number task, a black square with a side length of 20 mm served as the stimulus and was presented 12 cm to the left or the right of the screen center. Participants responded vocally by saying “one” or “two”.

#### Procedure

**Fig. 1:**
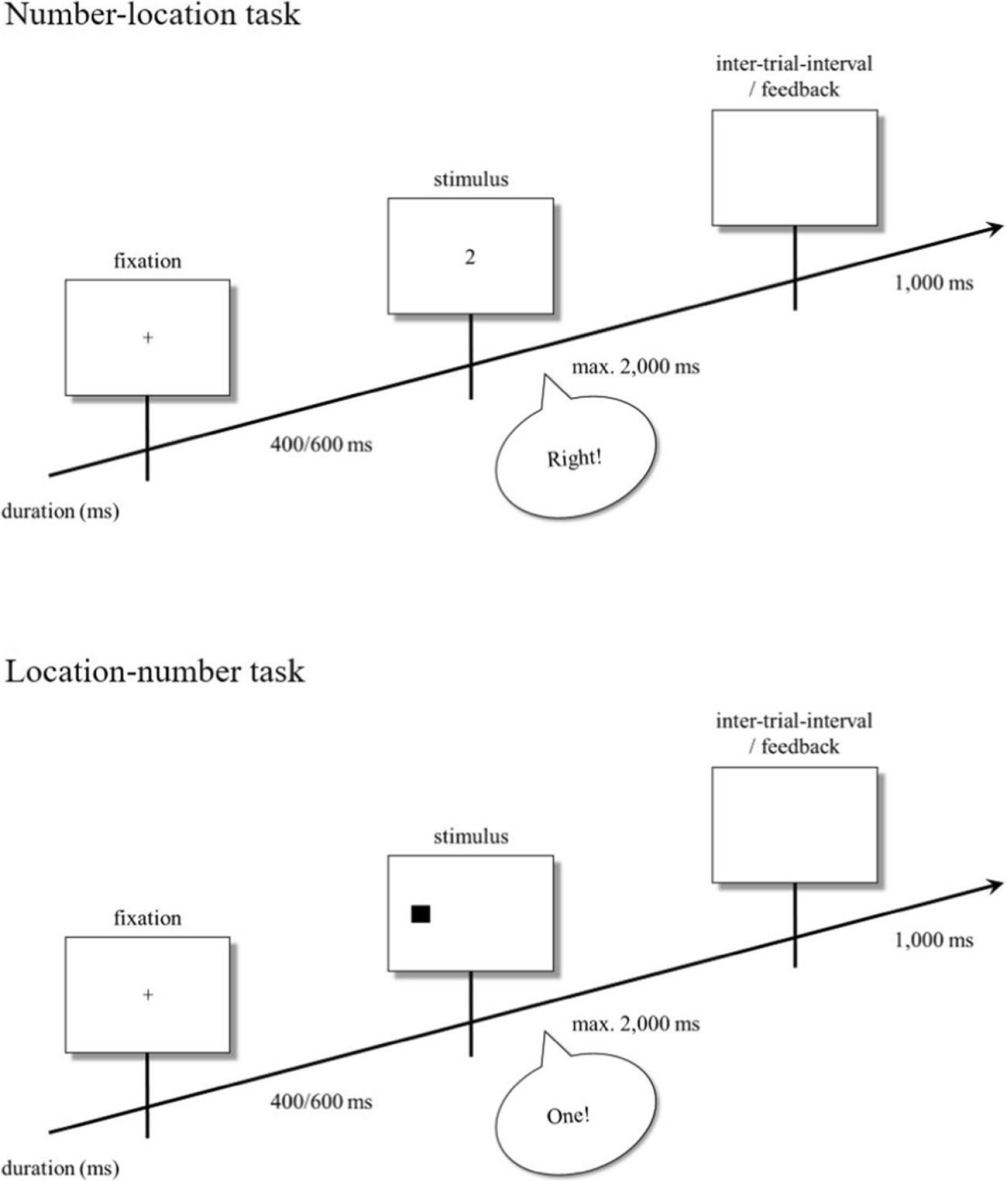
Time course of events in typical trials of the number–location task (upper panel), and the location–number task (lower panel) according to compatible mappings. Feedback was only provided after a missing response. The rectangles represent stimulus displays; the speech bubble represents a vocal response to the stimulus

Combining two tasks (number–location task, location–number task) and two S-R mappings (compatible, incompatible) resulted in four conditions which were completed by each participant. In the number–location task, participants responded vocally to the Arabic digits 1 or 2 by saying “left” or “right” according to a compatible mapping (1–“left”; 2–“right”) or an incompatible mapping (1–“right”; 2–“left”). In the location–number task, participants responded vocally to the left or right stimulus location by saying “one” or “two” according to a compatible mapping (left–“one”; right–“two”) or an incompatible mapping (left–“two”; right–“one”). The time course and sample stimuli of the number–location task and the location–number task are depicted in Fig. 1.

Instructions presented at the beginning of each condition informed participants about the content and the procedure of the task. Each condition consisted of one training block with 10 trials and two experimental blocks with 40 trials each. Within each block, trials were randomized. A fixation point was presented at the beginning of each trial for 400 or 600 ms, with both durations occurring equally often within each block. The stimulus was then presented until a response was registered or for a maximum of 2,000 ms. If the voice key registered a response, an intertrial interval showing an empty screen was presented for 1,000 ms. If the voice key did not register a response, a corresponding error message indicating a missing response was presented during the intertrial interval. Trials with a missing response were not repeated. Since the program could not identify the correctness of vocal responses, participants were not provided feedback about response accuracy. At the beginning of each experimental block, the instructions informing the participants about the task and S-R mapping were again presented. Participants were allowed to take a break between blocks or to continue with the subsequent one.

Both factors (i.e., task and S-R mapping) were varied within-subjects but between different blocks of trials. Both the order of tasks (number–location or location–number task first) and the order of mappings (compatible or incompatible mapping first) were counterbalanced between participants. Both S-R mapping conditions were completed consecutively within one task and the order of mappings was consistent between the two tasks for one participant. The whole experiment took about 30 min. The experimenter stayed in the laboratory during the practice blocks but left the room before the participants continued with the experimental blocks.

#### Design and data analysis

Before the statistical analysis, the vocal responses recorded during the experiment were checked in terms of accuracy for each participant. Response errors were manually entered into each data file. A response was counted as erroneous when the first sound could be ascribed to the wrong response despite potentially switching to the correct response subsequently. The design of the experiment was a two-factorial (Task × Mapping) within-subjects design. The first independent variable Task had two levels: the number–location task and the location–number task. The second factor S-R Mapping also contained two levels: the compatible mapping condition and the incompatible mapping condition.

We measured reaction times (RTs) of correct vocal responses and error percentages as dependent variables. We planned to analyze our data with a two-factorial analysis of variance (ANOVA), with Task (number–location, location–number) and S-R Mapping (compatible, incompatible) as within-subjects variables. In case of a significant two-way interaction, we planned to conduct *t* tests to determine the source of the interaction. Even though error percentages are usually not normally distributed which would indicate the use of nonparametric tests, we preferred using *t* tests due to the large number of ties in error percentages which are excluded from nonparametric tests and thus bias the results towards H1. For both directions, we use the label H1 (experimental hypothesis) to denote a significant effect and H0 (null hypothesis) to denote a nonsignificant effect. Moreover, we report the Bayes factor (BF) for each pairwise comparison (Rouder et al., 2009), which allows us to evaluate the evidence for H1 and H0. Assuming unidirectional associations between number and space implies a null effect in the location–number task. However, since absence of evidence is not evidence of absence, the null-hypothesis significance testing falls short in evaluating evidence for H0. We interpret the BF values according to the evidence categories provided by Jeffreys (1961; as cited in Lee & Wagenmakers, 2014).

Previous studies have shown that the size of compatibility or congruency effects such as the SNARC effect depends on response speed (i.e., RT; see Proctor et al., 2011, for a review). Regarding the regular SNARC effect in number–location tasks, it has been observed that the effect increases with increasing RTs (e.g., Gevers et al., 2006; Mapelli et al., 2003). We therefore conducted additional distributional analyses^4^ investigating the time course of the mapping effects in both the number–location task as well as the location–number task. With that we were able to check if the time course of the regular SNARC effect in our number–location task conforms to the time course of the SNARC effect which has been empirically established so far. Moreover, we wanted to rule out the possibility that a small mapping effect occurred for a specific RT level in the location–number task, which did not reach significance in the primary analysis.

### Results

#### Data trimming

On an overall level, we excluded one participant from data analysis (number 34 in our data set) because her mean error percentage in the number–location task was 20% or higher. After removing this dataset, the highest error percentages were 13.5% in the number–location task, and 6.1% in the location–number task. On a trial level, the first trial in each block and trials with RTs below 100 ms or above 1,500 ms were excluded from data analysis. Participants’ responses were too fast (i.e., RT < 100 ms) in less than 1% of trials in both the number–location task (*M* = 0.078%, *SD* = 0.311) and in the location–number task (*M* = 0.157%, *SD* = 0.585). Similarly, participants’ responses were too slow (i.e., RT > 1,500 ms) in less than 1% of trials in both the number–location task (*M* = 0.275%, *SD* = 0.817) and in the location–number task (*M* = 0.278%, *SD* = 1.114).

#### Reaction times (RTs)

We conducted a two-factorial ANOVA, with Task and Mapping as within-subjects factors and RTs from trials with correct responses as a dependent variable. Both main effects and the two-way interaction were significant. The significant main effect of Task, *F*(1, 58) = 71.367, *MSE* = 1,252.917, *p* < .001, η_p_^2^ = .552, indicated shorter RTs in the location–number task (*M* = 429 ms, *SD* = 66) than in the number–location task (*M* = 468 ms, *SD* = 74). The significant main effect of Mapping, *F*(1, 58) = 44.778, *MSE* = 817.958, *p* < .001, η_p_^2^ = .436, reflected shorter RTs with the compatible mapping (*M* = 436 ms, *SD* = 68) than with the incompatible mapping (*M* = 461 ms, *SD* = 76). Most interestingly, however, the two-way interaction, *F*(1, 58) = 20.311, *MSE* = 1,061.135, *p* < .001, η_p_^2^ = .259, was significant, revealing different mapping effects in the two tasks.

**Fig. 2:**
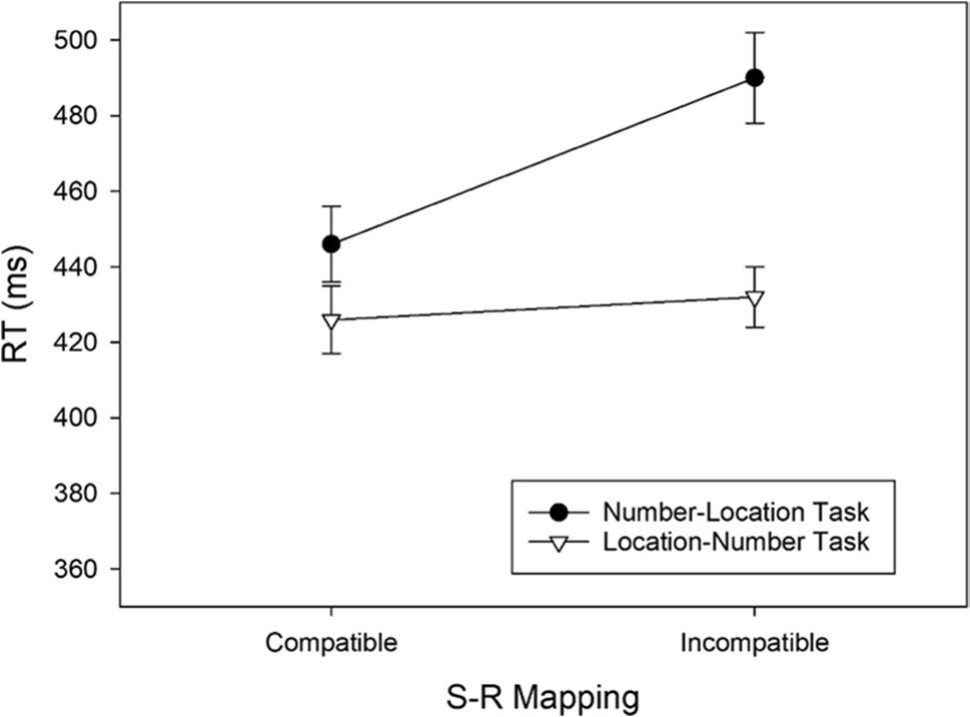
RTs of correct responses as a function of Task and S-R Mapping observed in Exp. 1. Error bars reflect 95% confidence intervals for within-subjects designs (Cousineau, 2017)

By means of pairwise comparisons between the compatible and incompatible mapping condition for each task we aimed to determine the source of the two-way interaction. In the number–location task, significantly shorter RTs in the compatible than in the incompatible condition, *t*(58) = 6.410, *p* < .001, *d* = 0.834, BF_+0_ = 454,889.170, revealed a regular SNARC effect of 44 ms (cf. Fig. 2) and extreme evidence for H1. Contrarily, in the location–number task, RTs did not differ significantly between both mapping conditions, *t*(58) = 1.428, *p* = .159, *d* = 0.186, BF_+0_ = 0.372, indicating anecdotal evidence against the presence of a reciprocal SNARC effect.

#### Error percentages

Even though error percentages were very low and the results of the statistical analysis should thus be interpreted with caution, we decided to report the analysis of error percentages for the sake of completeness. Importantly, error percentages were the highest in conditions in which RTs were the slowest, ruling out a potential speed–accuracy trade-off.

Error percentages were also subjected to a two-factorial ANOVA with Task and Mapping as within-subjects variables. Both main effects and the two-way interaction again reached significance. The main effect of Task, *F*(1, 58) = 17.388, *MSE* = 5.679, *p* < .001, η_p_^2^ = .231, indicated significantly more errors in the number–location task (*M* = 2.200, *SD* = 3.252) than in the location–number task (*M* = 0.906, *SD* = 1.696). The significant main effect of Mapping, *F*(1, 58) = 19.175, *MSE* = 6.050, *p* < .001, η_p_^2^ = .248, reflected more errors with the incompatible mapping (*M* = 2.254, *SD* = 2.901) than with the compatible mapping (*M* = 0.852, *SD* = 2.211). Again, the most interesting finding was the significant two-way interaction, *F*(1, 58) = 11.448, *MSE* = 4.712, *p* = .001, η_p_^2^ = .165, which revealed different mapping effects in the two tasks.

**Fig. 3:**
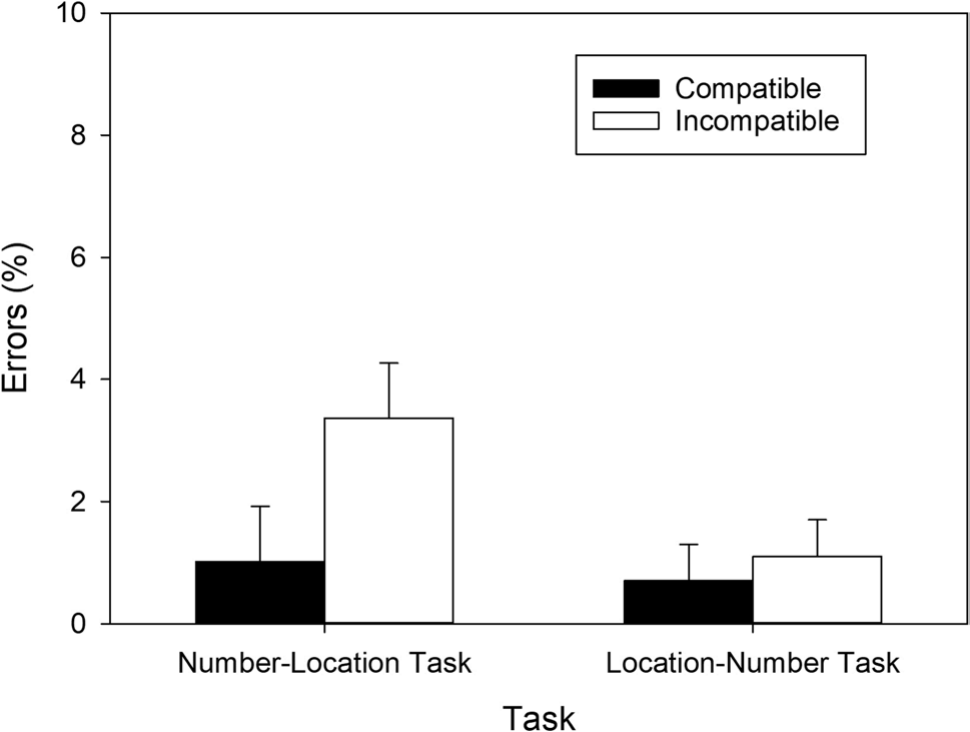
Error percentages as a function of Task and S-R Mapping observed in Exp. 1. Error bars reflect 95% confidence intervals for within-subjects designs (Cousineau, 2017)

To uncover the source of the two-way interaction, we again computed pairwise comparisons between the compatible and incompatible mapping condition for each task separately. In the number–location task, significantly fewer errors were made in the compatible than in the incompatible condition, *t*(58) = 4.423, *p* < .001, *d* = 0.576, BF_+0_ = 471.626, revealing a regular SNARC effect of 2.358% (cf. Fig. 3) and extreme evidence for H1. In contrast, in the location–number task, error percentages did not differ between the two mapping conditions, *t*(58) = 1.572, *p* = .121, *d* = 0.205, BF_+0_ = 0.454, revealing anecdotal evidence against the presence of a reciprocal SNARC effect.

#### Exclusion of outliers

Reciprocal SNARC effects did not reach significance in the omnibus analysis or the distributional analysis (for the latter, see the Appendix). Nevertheless, small numerical trends of up to 17 ms (in the fourth quartile of the distributional analysis) emerged in the location–number task. Since a previous study has shown that outliers might be driving these small reciprocal SNARC effects (Richter & Wühr, 2023), we decided to run the same set of analysis after excluding outlier participants according to the Tukey (1977) criterion, which identifies observations below Q_25_ – 1,5×IQR or above Q_75_ + 1,5×IQR as outliers. We collapsed data across the mapping variable and applied the criterion to four variables (mean RT and error percentage in the number–location and location–number task, respectively), which led to the exclusion of eight further participants and a remaining sample of *N* = 51. By removing outliers, the numerical trends of small reciprocal SNARC effects in RTs decreased from 6 ms (*d* = 0.186) to 3 ms (*d* = 0.111) in the pairwise comparisons, while evidence for the null hypothesis increased from anecdotal (BF_+0_ = 0.372) to moderate (BF_+0_ = 0.206). The same pattern was observable for numerical trends in error percentages, which decreased from 0.446% (*d* = 0.205) to 0.104% (*d* = 0.078), while evidence for the null hypothesis increased from anecdotal (BF_+0_ = 0.454) to moderate (BF_+0_ = 0.117).

### Discussion

The purpose of Experiment 1 was to investigate the reciprocity of the SNARC effect. Therefore, we compared the compatibility effect of a number–location task (SNARC task) with the compatibility effect of a location–number task (reciprocal SNARC task). As expected, we found a regular SNARC effect in the number–location task. Participants’ vocal responses were faster and more accurate in the compatible mapping condition (1–“left”; 2–“right”) than in the incompatible mapping condition (1–“right”; 2–“left”). Moreover, the SNARC effect in RTs showed the typical time course as it increased with increasing RTs (e.g., Gevers et al., 2006; Mapelli et al., 2003). In contrast, we did not find a reciprocal SNARC effect in the location–number task. An additional distributional analysis (see Appendix) corroborated this finding: although the two-way interaction of Mapping and Quartile was significant for this task, suggesting a numerical increase of reciprocal SNARC effects with increasing RTs, post hoc tests could not detect a significant mapping effect for any quartile. Our results thus indicate that digit stimuli activated vocal location responses, whereas spatial stimuli did not activate vocal number responses with the same strength. The finding that small numerical trends of reciprocal SNARC effects increased with increasing RTs might at first glance suggest that long RTs are required for reciprocal SNARC effects to emerge. However, excluding outliers eliminated this interaction implying that small reciprocal SNARC effects were mainly driven by a small subset of participants who showed extreme RTs and/or error percentages. Nevertheless, spatial–numerical associations of vocal response codes seem to be at least strongly asymmetrical.

## Experiment 2

Experiment 2 served two aims. The first aim was to replicate the pattern of results of Experiment 1 with a different stimulus set and a different sample. Therefore, we used the numerical values 1 and 9 as digit stimuli in the number–location task, and as (vocal) number responses in the location–number task. The second aim was to test how removing a possible influence of the so-called *markedness association of response codes* (MARC) effect from the design would affect the pattern of results. The MARC effect refers to faster left-hand responses to odd digit stimuli, and faster right-hand responses to even digit stimuli, when compared with the reverse combinations (e.g., Berch et al., 1999; Cipora et al., 2019; Nuerk et al., 2004). In Experiment 1, MARC compatibility and SNARC compatibility were confounded because the small number (1) was odd, and the larger number (2) was even. Therefore, we cannot exclude that the mapping effect observed in the number–location task reflected a combination of both SNARC and MARC effects. Similarly, the small compatibility effects in the location–number task, which were observed for longer RTs, might either reflect reciprocal SNARC effects, reciprocal MARC effects, or both. In Experiment 2, we wanted to isolate the asymmetrical pattern for the SNARC effect by excluding any contribution of the MARC effect. This was achieved by using two odd numbers (1 and 9) as the small and the large number.

### Methods^5^

#### Participants

We applied the same rationale as in Experiment 1 to estimate the effect size and conduct a power analysis. Therefore, again, a sample size of 54 participants would be required to detect an effect of η_p_^2^ = 0.2 with high power (1 − β = .95) at the standard alpha-error probability of .05.

Sixty-one students (42 female, 19 male) with a mean age of 23.262 years (*SD* = 3.723) volunteered in our experiment. According to self-report, 54 participants were right-handed and seven participants were left-handed. All participants reported to have normal (*N* = 40) or corrected-to-normal (*N* = 21) vision. All volunteers gave their informed consent prior to participation and received either course credit or a payment of 10 Euro in exchange. Again, the local Ethics Committee at TU Dortmund University approved the experimental protocol for our study (GEKTUDO_2022_36).

#### Apparatus and stimuli

The apparatus and stimuli were the same as in Experiment 1, with the only exception that we replaced the digit stimuli 1 / 2 and the corresponding number responses “one”/“two”, which were used in Experiment 1, by the digit stimuli 1 / 9 and the number responses “one”/“nine”. Thus, in the number–location task, the Arabic digits 1 or 9 served as stimuli to which participants responded vocally by saying “left” or “right”, whereas in the location–number task, a black square occurred to the left or right of fixation, and participants responded vocally to stimulus location by saying “one” or “nine”.

#### Procedure

The procedure in Experiment 2 was the same as in Experiment 1 with the only exception that different numerical stimuli and responses were employed. In the number-location task, the compatible mapping thus contained the assignments 1–“left”/9–“right” and the incompatible mapping contained the assignments 1–“right”/9–“left”. In the location–number task, the compatible mapping consisted of the assignments left–“one”/right–“nine” and the incompatible mapping consisted of the assignments left–“nine”/right–“one”.

#### Design and data analysis

The design and data analysis were the same as in Experiment 1.

### Results

#### Data trimming

On an overall level, we excluded three participants due to technical difficulties.^6^ On a trial level, the first trial in each block and trials with RTs below 100 ms or above 1,500 ms were excluded from data analysis. Participants’ responses were too fast (i.e., RT < 100 ms) in less than 1% of trials in both the number–location task (*M* = 0.079%, *SD* = 0.399) and in the location–number task (*M* = 0.078%, *SD* = 0.351). Similarly, participants’ responses were too slow (i.e., RT > 1,500 ms) in less than 1% of trials in both the number–location task (*M* = 0.236%, *SD* = 0.807) and in the location–number task (*M* = 0.236%, *SD* = 0.876).

#### Reaction times (RTs)

We conducted a two-factorial ANOVA, with Task and Mapping as within-subjects factors and RTs from trials with correct responses as a dependent variable. Both main effects and the two-way interaction were significant. The significant main effect of Task, *F*(1, 57) = 228.505, *MSE* = 860.703, *p* < .001, η_p_^2^ = .800, reflected shorter RTs in the location–number task (*M* = 387 ms, *SD* = 59) than in the number–location task (*M* = 445 ms, *SD* = 68). The significant main effect of Mapping, *F*(1, 57) = 11.355, *MSE* = 1,080.322, *p* = .001, η_p_^2^ = .166, indicated shorter RTs with the compatible mapping (*M* = 409 ms, *SD* = 59) than with the incompatible mapping (*M* = 423 ms, *SD* = 79). Most interestingly, however, the two-way interaction, *F*(1, 57) = 6.208, *MSE* = 841.351, *p* = .016, η_p_^2^ = .098, was significant, indicating different mapping effects in the two tasks.

**Fig. 4:**
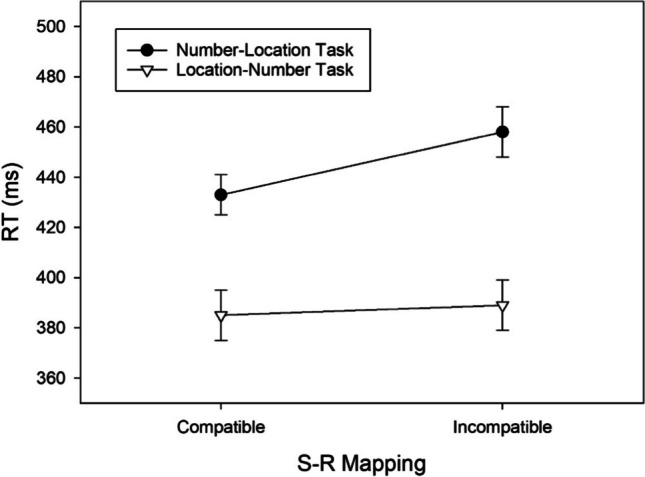
RTs of correct responses as a function of Task and S-R Mapping observed in Exp. 2. Error bars reflect 95% confidence intervals for within-subjects designs (Cousineau, 2017)

In the number–location task, significantly shorter RTs in the compatible than in the incompatible condition, *t*(57) = 4.416, *p* < .001, *d* = 0.580, BF_+0_ = 454.621, revealed a regular SNARC effect of 24 ms (cf. Fig. 4) and extreme evidence for H1. Contrarily, in the location–number task, RTs did not differ significantly between both mapping conditions, *t*(57) = 0.835, *p* = .407, *d* = 0.110, BF_+0_ = 0.200, indicating moderate evidence against the presence of a reciprocal SNARC effect.

#### Error percentages

We again decided to report the analysis of error percentages, even though error percentages were very low and the results of the statistical analysis should thus be interpreted with caution. Similar to Experiment 1, error percentages were the highest in conditions in which RTs were the slowest, ruling out a potential speed–accuracy trade-off.

Error percentages were subjected to a two-factorial ANOVA, with Task and Mapping as within-subjects variables. Both main effects and the two-way interaction were again significant. The main effect of Task, *F*(1, 57) = 24.011, *MSE* = 1.796, *p* < .001, η_p_^2^ = .296, indicated significantly more errors in the number–location task (*M* = 1.538, *SD* = 2.251) than in the location–number task (*M* = 0.676, *SD* = 1.212). The significant main effect of Mapping, *F*(1, 57) = 9.283, *MSE* = 3.455, *p* = .004, η_p_^2^ = .140, reflected more errors with the incompatible mapping (*M* = 1.479, *SD* = 2.306) than with the compatible mapping (*M* = 0.735, *SD* = 1.148). Again, the most interesting finding was the significant two-way interaction, *F*(1, 57) = 16.051, *MSE* = 1.924, *p* < .001, η_p_^2^ = .220, which revealed different mapping effects in the two tasks.

**Fig. 5:**
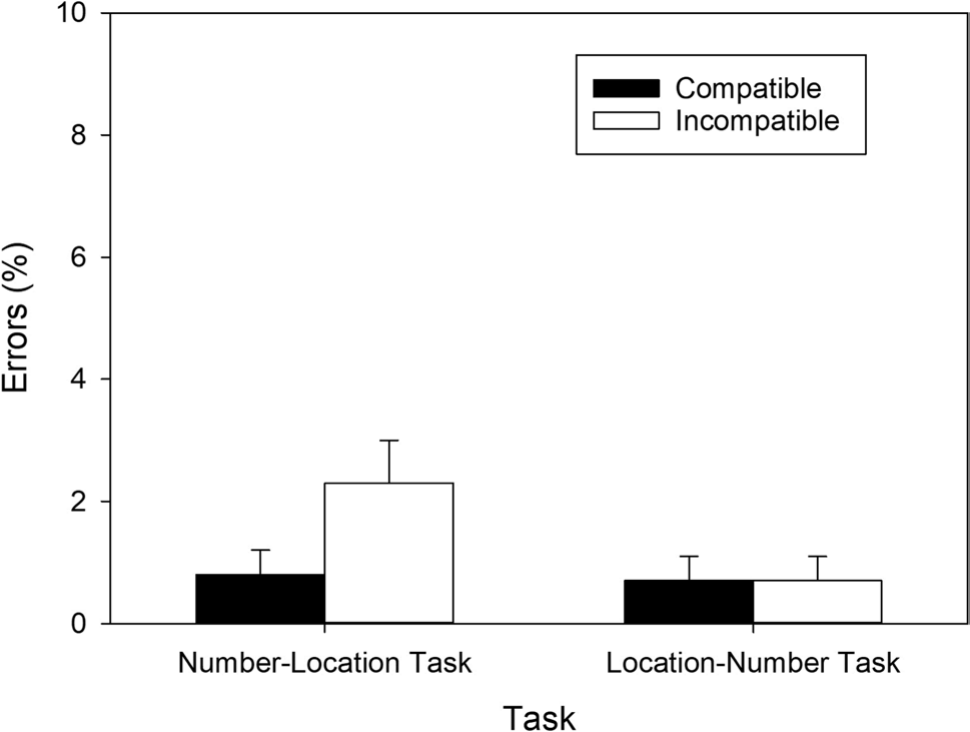
Error percentages as a function of Task and S-R Mapping observed in Exp. 2. Error bars reflect 95% confidence intervals for within-subjects designs (Cousineau, 2017)

In the number–location task, significantly fewer errors were made in the compatible than in the incompatible condition, *t*(57) = 3.843, *p* < .001, *d* = 0.505, BF_+0_ = 78.679, revealing a regular SNARC effect of 1.473% (cf. Fig. 5) and very strong evidence for H1. In contrast, in the location–number task, error percentages did not differ between the two mapping conditions, *t*(57) = 0.071, *p* = .944, *d* = 0.009, BF_+0_ = 0.144, indicating moderate evidence against the presence of a reciprocal SNARC effect.

#### Exclusion of outliers

Again, we conducted the same set of analysis after excluding outlier participants. Applying the Tukey (1977) criterion led to the exclusion of four participants and a remaining sample of *N* = 54. By removing outliers, the numerical trends of small reciprocal SNARC effects in RTs decreased from 5 ms (*d* = 0.110) to <1 ms (*d* = 0.004) in the pairwise comparisons, consistently providing moderate evidence against the presence of a reciprocal SNARC effect (BF_+0_ = 0.148).

### Discussion

The results of Experiment 2 replicated the main findings of Experiment 1 with a different stimulus and response set. We found a regular SNARC effect in the number–location task of Experiment 2, and no reciprocal SNARC effect in the location–number task. The time course pattern of the regular SNARC effect was again congruent with the one reported before (e.g., Gevers et al., 2006; Mapelli et al., 2003; see Appendix). Numerically small trends of reciprocal SNARC effects disappeared completely after excluding outliers, which thus supports the hypothesis that bidirectional SNARC effects might occur for a small subsample showing extreme RTs and/or error percentages. Since two odd numbers (1, 9) were used as stimuli and responses in Experiment 2, we excluded a potential impact of the MARC effect on our results, and the mapping effects observed can be interpreted as pure SNARC effects. The result that a regular but no reciprocal SNARC effect occurred despite the absence of a MARC effect strengthens the finding of Experiment 1 that the spatial–numerical associations, which underlie SNARC effects, are strongly asymmetrical for the vocal response mode.

## Experiment 3

The major aim of Experiment 3 was to test how a consistent use of alphanumeric stimuli and responses would affect the pattern of results. In Experiments 1 and 2, we used alphanumeric stimuli (1 / 2 or 9) and responses (“left”/“right”) in the number–location task. In the reciprocal location–number task, we also used alphanumeric responses (“one”/“two” or “nine”), but we used visuospatial stimuli (left and right physical locations). The change of the stimulus presentation mode from alphanumeric to visuospatial makes the location–number task less comparable with the number–location task for two reasons. Firstly, while attention remains concentrated on the fixation point in the number–location task, it is allocated to the left or right in the location–number task. Secondly, the visuospatial instead of the verbal-spatial coding (cf. Gevers et al., 2010) might be emphasized in the location–number task but not in the number–location task. The asymmetry of spatial-size associations which we found in Experiments 1 and 2 could therefore also be attributed to asymmetrical associations of stimulus codes instead of response codes. In Experiment 3, we replaced the visuospatial stimuli in the location–number task by alphanumeric stimuli in the form of location words (“left”/“right”). For consistency reasons, we also replaced the digits, which we employed as stimuli in the number–location task, by number words (“one”/“nine”).

### Methods

#### Participants

We aimed to attain a similar power as in Experiments 1 and 2. Fifty-two students (35 female, 17 male) with a mean age of 23.712 years (*SD* = 3.177) volunteered in our experiment. According to self-report, 45 participants were right-handed and seven participants were left-handed. All participants reported to have normal (*N* = 27) or corrected-to-normal (*N* = 25) vision. All volunteers gave their informed consent prior to participation and received either course credit or a payment of 10 Euro in exchange. Again, the local Ethics Committee at TU Dortmund University approved the experimental protocol for our study (GEKTUDO_2022_36).

#### Apparatus and stimuli

The apparatus and stimuli were the same as in Experiment 2, with the only exception that we replaced the numerical digits 1 / 9 by the number words “one”/“nine” in the number–location task and the left/right square stimuli by the centrally presented location words “left”/“right” in the location–number task. All stimuli were presented in 40 pt in Times New Roman. Thus, in the number–location task, the words “one” or “nine” served as stimuli to which participants responded vocally by saying “left” or “right”, whereas in the location–number task, the words “left” or “right” served as stimuli to which participants responded by saying “one” or “nine”.

#### Procedure

The procedure in Experiment 3 was the same as in Experiment 2, with the only exception that different stimuli were employed. In the number-location task, the compatible mapping thus contained the assignments “one”–“left”/“nine”–“right” and the incompatible mapping contained the assignments “one”–“right”/“nine”–“left”. In the location–number task, the compatible mapping consisted of the assignments “left”–“one”/“right”–“nine” and the incompatible mapping consisted of the assignments “left”–“nine”/“right”–“one”.

#### Design and data analysis

The design and data analysis were the same as in Experiments 1 and 2. Moreover, we conducted a comparison of mapping effects between experiments^7^ to investigate in how far the changes between experiments affected the regular and the reciprocal SNARC effects, respectively.

### Results

#### Data trimming

On an overall level, we excluded two participants (21 and 48 in our dataset) because their mean error percentage in the location–number task was 20% or higher. On a trial level, the first trial in each block and trials with RTs below 100 ms or above 1,500 ms were excluded from data analysis. Participants’ responses were too fast (i.e., RT < 100 ms) in less than 1% of trials in both the number–location task (*M* = 0.077%, *SD* = 0.307) and in the location–number task (*M* = 0.130%, *SD* = 0.632). Similarly, participants’ responses were too slow (i.e., RT > 1,500 ms) in less than 1% of trials in both the number–location task (*M* = 0.237%, *SD* = 1.179) and in the location–number task (*M* = 0.180%, *SD* = 0.709).

#### Reaction times (RTs)

We conducted a two-factorial ANOVA, with Task and Mapping as within-subjects factors and RTs from trials with correct responses as a dependent variable. Only the main effect of Mapping, *F*(1, 49) = 11.532, *MSE* = 2,807.828, *p* = .001, η_p_^2^ = .191, was significant indicating shorter RTs with the compatible mapping (*M* = 486 ms, *SD* = 62) than with the incompatible mapping (*M* = 512 ms, *SD* = 80). The main effect of Task, *F*(1, 49) = 0.651, *MSE* = 1,897.706, *p* = .424, η_p_^2^ = .013, and the two-way interaction, *F*(1, 49) = 0.382, *MSE* = 1,427.844, *p* = .539, η_p_^2^ = .008, were nonsignificant, indicating similar RT levels and similar mapping effects in the two tasks.

**Fig. 6:**
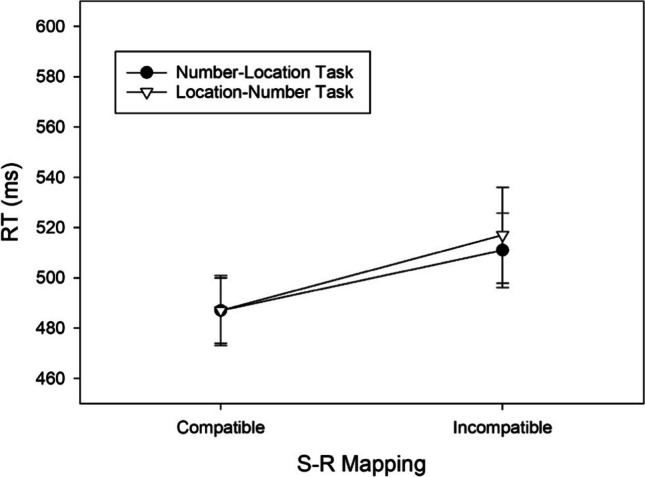
RTs of correct responses as a function of Task and S-R Mapping observed in Exp. 3. Error bars reflect 95% confidence intervals for within-subjects designs (Cousineau, 2017)

In the number–location task, significantly shorter RTs in the compatible than in the incompatible condition, *t*(49) = 2.673, *p* = .01, *d* = 0.378, BF_+0_ = 3.700, revealed a regular SNARC effect of 22 ms (cf. Fig. 6) and moderate evidence for H1. In the location–number task, significantly shorter RTs in the compatible than in the incompatible condition, *t*(49) = 2.864, *p* = .006, *d* = 0.405, BF_+0_ = 5.723, revealed a reciprocal SNARC effect of 29 ms and moderate evidence for H1.

#### Error percentages

Error percentages were subjected to a two-factorial ANOVA, with Task and Mapping as within-subjects variables. The main effect of Task, *F*(1, 49) = 2.781, *MSE* = 3.869, *p* = .102, η_p_^2^ = .054, was nonsignificant. The main effect of mapping and the two-way interaction were significant. The significant main effect of Mapping, *F*(1, 49) = 12.776, *MSE* = 10.094, *p* < .001, η_p_^2^ = .207, reflected more errors with the incompatible mapping (*M* = 4.303, *SD* = 3.245) than with the compatible mapping (*M* = 2.697, *SD* = 2.931). The significant two-way interaction, *F*(1, 49) = 4.501, *MSE* = 2.710, *p* = .039, η_p_^2^ = .084, revealed differences in the mapping effects between the two tasks.

**Fig. 7:**
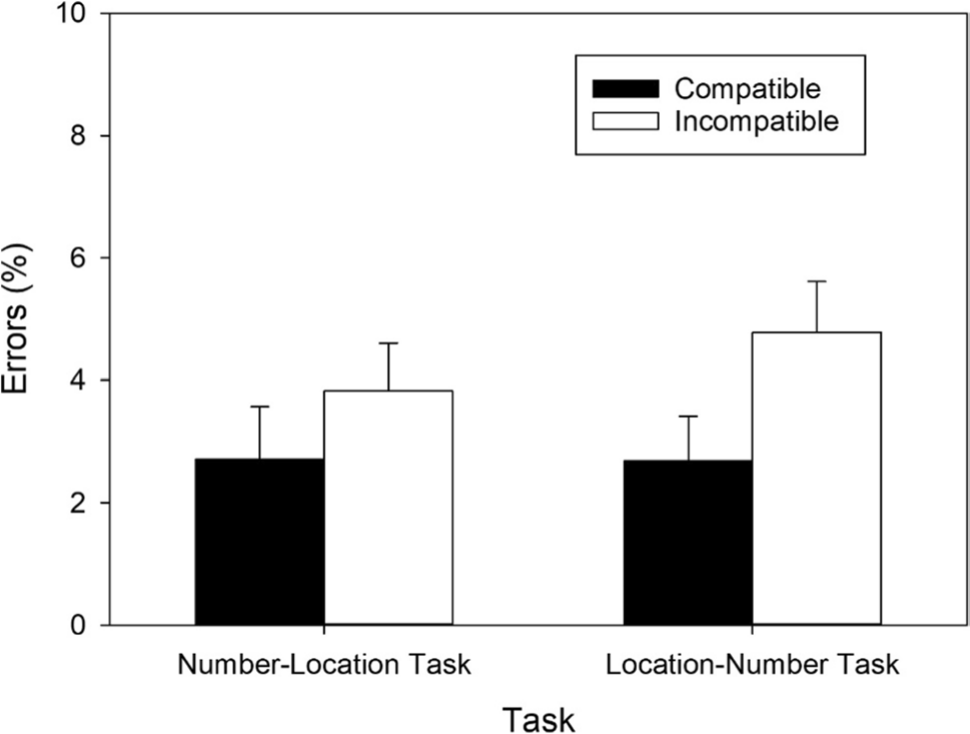
Error percentages as a function of Task and S-R Mapping observed in Exp. 3. Error bars reflect 95% confidence intervals for within-subjects designs (Cousineau, 2017)

In the number–location task, significantly fewer errors were made in the compatible than in the incompatible condition, *t*(49) = 2.129, *p* = .038, *d* = 0.301, BF_+0_ = 1.214, revealing a regular SNARC effect of 1.112% (cf. Fig. 7) and anecdotal evidence for H1. In the location–number task, significantly fewer errors were made in the compatible than in the incompatible condition, *t*(49) = 4.292, *p* < .001, *d* = 0.607, BF_+0_ = 271.313, indicating a reciprocal SNARC effect of 2.1% (cf. Fig. 7) and extreme evidence for H1.

#### Exclusion of outliers

Again, we conducted the same set of analysis after excluding outlier participants. Applying the Tukey (1977) criterion led to the exclusion of four participants and a remaining sample of *N* = 46. In the omnibus analysis of error percentages without outliers, the two-way interaction Mapping × Task, *F*(1, 45) = 3.015, *MSE* = 2.769, *p* = .089, η_p_^2^ = .063, became nonsignificant indicating similar mapping effects in the two tasks.

### Discussion

The results of Experiment 3 were quite different from the results of Experiments 1 and 2. We again obtained a regular SNARC effect in the number–location task, but this time we also obtained a reciprocal SNARC effect in the location–number task: Participants’ responses were faster and more accurate when they responded to the location word “left” by saying “one” and to the location word “right” by saying “nine” as compared with the reverse assignment. In terms of size, the reciprocal SNARC effect was similar to the regular SNARC effect for RTs and larger than the regular SNARC effect for error percentages. The distributional analyses (see Appendix) revealed similar time courses for the regular and reciprocal SNARC effect, which both increased with increasing RTs. Excluding outliers merely affected the differences between the regular and reciprocal SNARC effect, which vanished for error percentages and emerged for the time courses, but did not affect the emergence of a reciprocal SNARC effect itself. Hence, we observed a reciprocal SNARC effect when verbal stimuli and vocal responses were used in the location–number task, which was absent when visuospatial stimuli (i.e., locations) were used in combination with manual responses (Richter & Wühr, 2023) or in combination with vocal responses (Experiments 1 and 2). Alphanumeric stimuli thus seem to foster the occurrence of a reciprocal SNARC effect. The finding that regular and reciprocal SNARC effects were of similar size with alphanumeric stimuli suggests that the spatial–numerical associations, which underlie the SNARC effects in these tasks, are bidirectional and symmetrical in this particular case.

A comparison of mapping effects between experiments (see Appendix), moreover, revealed that the regular SNARC effect decreased from Experiment 1 to Experiments 2 and 3, which can be attributed to the employment of the numerical values 1 and 9 in both latter experiments and the employment of numerical 1 and 2 in the former experiment. Contrarily, the reciprocal SNARC effect increased from Experiments 1 and 2 to Experiment 3, which can be attributed to the employment of verbal compared with visuospatial stimuli.

## General discussion

We conducted three experiments that directly compared SNARC effects in a number–location task to reciprocal SNARC effects in a location–number task with vocal responses in both tasks. In all three experiments, we found a regular SNARC effect in the number–location task with alphanumeric stimuli and responses. Participants’ responses were faster and more accurate when they responded to a small number by saying “left” and to a large number by saying “right” as compared with the reverse assignment. This pattern emerged regardless of using digits (Experiments 1 and 2) or number words (Experiment 3) as stimuli. Additional distributional analysis revealed that the SNARC effect increased with increasing RTs, resembling the typical time course pattern of SNARC effects (e.g., Gevers et al., 2006; Mapelli et al., 2003).

In contrast, we did not find a reciprocal SNARC effect in the location–number task of Experiments 1 and 2, in which we used visuospatial stimuli (left/right physical locations). Regardless of RT level, response times and error percentages did not differ significantly between the compatible and the incompatible mapping condition. In contrast, we did find a reciprocal SNARC effect in the location–number task of Experiment 3, in which we used verbal stimuli (i.e., location words “left”/“right”) and vocal responses. Participants’ responses were faster and more accurate when they responded to the location word “left” by saying “one” and to the location word “right” by saying “nine” as compared with the reverse assignment. When using alphanumeric stimuli and responses, regular and reciprocal SNARC effects were of similar size. Thus, location words but not physical locations can influence the selection and execution of vocal number responses to a similar extent as number words can influence the selection and execution of vocal location responses.

Together with the results of a previous study with manual responses (Richter & Wühr, 2023), the following empirical picture emerges. The regular SNARC effect occurred for all different combinations of stimulus and response sets: We observed the regular SNARC effect with numerosity stimuli and manual keypress responses, digit stimuli and manual keypress responses, digit stimuli and vocal responses, and with number word stimuli and vocal responses. These results confirm previous studies that have demonstrated regular SNARC effects of similar size with different stimulus modes (e.g., Nuerk et al., 2005) and different response modes (i.e., manual and vocal; Gevers et al., 2010).

The reciprocal SNARC effect, in contrast, varied considerably with different stimulus and response modes. With visuospatial stimuli and manual number responses (Richter & Wühr, 2023), and visuospatial stimuli and vocal number responses (present Experiments 1 and 2), we observed very weak reciprocal SNARC effects that disappeared when outlier data sets were excluded. Hence, for these combinations of stimulus and response sets, regular SNARC effects were much stronger than reciprocal SNARC effects suggesting asymmetrical S-R associations. Only with verbal stimuli and vocal responses did we observe regular and reciprocal SNARC effects of similar size (Experiment 3).

Some studies suggest that the symmetry or asymmetry of spatial–numerical associations might depend on response mode: While spatiomotor responses tend to cause much interference between space and number (Cona et al., 2021; Decarli et al., 2022; Lindemann et al., 2007), thus potentially fostering symmetrical associations, vocal tasks on the contrary might foster asymmetrical associations (Walsh, 2013). Our observation of symmetrical SNARC effects with alphanumeric stimuli and responses but asymmetrical SNARC effects with non-alphanumeric stimuli and responses, however, points towards the opposite hypothesis. To further investigate potential asymmetries between patterns of data using motor versus vocal responses, we compared the results of Experiment 2 of our previous work (Richter & Wühr, 2023), where we used manual responses, to the results of Experiment 1 of our present work where we used the same stimuli but vocal responses. Importantly, there were no differences in the regular SNARC effect between response modes. Moreover, there were no differences in the reciprocal SNARC effect between response modes. Response mode therefore did not affect regular or reciprocal SNARC effects in our experiments.

The finding of bidirectional and symmetrical associations between number and space in S-R priming tasks employing alphanumeric stimuli and responses is in line with spatial–numerical associations in general for which reciprocity has already been demonstrated. While bidirectional spatial–numerical associations have already been observed as stimulus–stimulus (S-S) congruency effects in priming tasks (Kramer et al., 2011; Stoianov et al., 2008) or as response–response (R-R) effects in random number generation tasks (Loetscher et al., 2008, 2010; Shaki & Fischer, 2014), the current experiments extend those findings by demonstrating that bidirectional spatial–numerical associations may under certain circumstances also emerge as stimulus–response (S-R) effects—that is, in the form of reciprocal SNARC effects.

### Possible sources for the occurrence of reciprocal SNARC effects

Several methodological aspects might have contributed to the observed variation in the occurrence of the reciprocal SNARC effect in our experiments. First, one might argue that set-level compatibility (e.g., Kornblum et al., 1990) was higher between verbal stimuli and vocal responses than for other combinations of stimulus and response sets, and higher set-level compatibility might have contributed to the occurrence of a reciprocal SNARC effect in Experiment 3. The findings that overall RTs were longer in Experiment 3 than in Experiment 2, and that the regular SNARC effect was of comparable size in the two experiments, however, provides evidence against an important role of set-level compatibility. Second, using verbal stimuli might have induced verbal-spatial coding, rather than visuospatial coding, and verbal coding of space might foster the occurrence of the reciprocal SNARC effect (cf. Gevers et al., 2010, for discussing the role of verbal-spatial coding for the regular SNARC effect). Third, the overall RT level was much higher with verbal than with visuospatial stimuli. Mean RTs in the location–number task increased from 387 ms in Experiment 2 (429 ms in Exp. 1) to 502 ms in Experiment 3. In line with the finding that reciprocal SNARC effects increase with increasing RTs, the emergence of reciprocal SNARC effects with verbal location stimuli might be mediated by higher RTs. Future research should address which factors are responsible for the emergence of reciprocal SNARC effects.

Nevertheless, small numerical trends of a reciprocal SNARC effect emerged for visuospatial stimuli, which significantly increased with increasing RTs. Even though the exclusion of outlier data eliminated those trends, we thus cannot exclude that reciprocal SNARC effects might occur for visuospatial stimuli under certain circumstances, in particular for participants who show high RTs and/or error percentages. This finding is consistent with the results of our previous study with manual responses (Richter & Wühr, 2023).

### Theoretical accounts of the SNARC effect

The theoretical accounts which have been proposed to explain SNARC effects differ in whether they predict bidirectional associations between number and space or not. Moreover, they differ in whether they can explain that stimulus mode affects the emergence of reciprocal but not regular SNARC effects. The results of our experiments are thus only in line with some of those accounts.

#### MNL

As one of the most prominent accounts of the SNARC effect, the MNL (e.g., Dehaene, 2003; Dehaene et al., 1993; Fischer & Shaki, 2014) assumes that numbers are spatially represented in ascending order from left to right. The MNL proposes a shared representation of number and space and thus the simultaneous activation of spatial and numerical information (e.g., Fischer & Shaki, 2015; Hartmann et al., 2012; Lugli et al., 2013; Shaki & Fischer, 2014; Stoianov et al., 2008). Yet the MNL account also assumes that this shared representation is located on an intermediate (semantic) level between stimulus processing and response selection (Ginsburg & Gevers, 2015; Huber et al., 2016; Umiltà et al., 2010). The asymmetry we observed in Experiments 1 and 2, and in our previous study (Richter & Wühr, 2023) could have thus occurred either between stimuli and the MNL, or between the MNL and responses. The observation that employing verbal location stimuli instead of visuospatial stimuli in the location–number task leads to symmetrical associations meanwhile suggests that stimulus mode is responsible for the asymmetry formerly observed. Verbal location stimuli thus seem to activate the MNL more strongly than visuospatial stimuli. This, however, seems to be at odds with the fact that the MNL itself constitutes a visuospatial account of spatial–numerical associations (Umiltà et al., 2010). In conclusion, the MNL account is flexible enough to account for symmetrical or asymmetrical patterns of SNARC and reciprocal SNARC effects, although it does not provide a direct answer for the observed impact of stimulus mode on the reciprocal SNARC effect.

#### Polarity correspondence principle

The polarity correspondence account of the SNARC effect assumes that negative polarities are assigned to “left” and “small”, whereas positive polarities are assigned to “large” and “right” (e.g., Proctor & Cho, 2006). Since polarities are attributes of dimensions, polarity coding of left-right and small-large should occur regardless of whether these dimensions vary on the stimulus or on the response side. Therefore, polarity coding should not only occur in our number–location task, which resembles the typical SNARC task, but also in our location–number task. In other words, according to our interpretation of the polarity correspondence account, it should predict reciprocal and symmetrical compatibility effects in the two tasks.

The predictions of the polarity correspondence account are consistent with the results of Experiment 3, where we observed similar SNARC and reciprocal SNARC effects with verbal (number or location) stimuli and vocal (location or number) responses. In contrast, the polarity correspondence account is not consistent with the results of the present Experiments 1 and 2, where we failed to observe reciprocal SNARC effects with physical location stimuli and vocal number responses. Similarly, the results of our previous experiments (Richter & Wühr, 2023), where we failed to observe a reciprocal SNARC effect with physical location stimuli and manual number responses, are also at odds with polarity correspondence. To account for the absence of reciprocal SNARC effects in these experiments, the polarity correspondence account would have to claim that, in these experiments, polarity coding did not occur for either the physical location stimuli or for the number responses. The fact that (vocal) number responses produced reciprocal SNARC effects in the present Experiment 3 falsifies the latter hypothesis. Hence, the polarity correspondence account must attribute the failure to obtain reciprocal SNARC effects in all the other experiments to the absence of polarity coding for physical location stimuli. This account, however, is implausible because polarity coding of location stimuli has been invoked in previous studies of picture-word verification (e.g., Just & Carpenter, 1975; Olson & Laxar, 1973) and orthogonal S-R correspondence effects (e.g., Cho & Proctor, 2003; Weeks & Proctor, 1990).

#### WM account

Lastly, the WM account proposes that the association between the serial position of a numerical stimulus stored in WM and the spatial location of the response leads to the (regular) SNARC effect (e.g., van Dijck & Fias, 2011; van Dijck et al., 2015). This account was later extended to, or incorporated into, a theory of coding the serial order of items in verbal WM, called the mental whiteboard hypothesis (e.g., Abrahamse et al., 2014, 2017; De Belder et al., 2015). According to this hypothesis, coding the serial order of items in (verbal) WM is achieved by connecting the items to spatial position markers. Since the spatial coding of serial order is assumed to occur from left to right, it allows to account for the SNARC effect with number stimuli and spatial responses (e.g., Abrahamse et al., 2016). In order to explain the regular SNARC effect in our task, proponents of the WM account would assume that participants (i) spontaneously represent the two number stimuli in an ascending order, which (ii) is achieved by connecting the smaller number (e.g., 1) to a left position marker and the larger number (e.g., 2) to a right position marker. The congruency, or incongruency, between the spatial position markers and the spatial responses then produces the observed SNARC effect (cf. Abrahamse et al., 2014).

Although the mental whiteboard hypothesis assumes bidirectional effects between WM retrieval and spatial processing (e.g., De Belder et al., 2015), it is not clear whether this account would predict (or explain) reciprocal SNARC effects. If we adapt the WM account of regular SNARC effects to our reciprocal SNARC task, we would have to assume that participants (i) spontaneously represent the two location stimuli in a canonical (i.e., left-to-right) order and (ii) connect the two stimuli to spatial position markers. But how should these position markers then prime the (manual or vocal) number responses that are required in this task? To our knowledge, the WM account does not consider the possibility that responses are also (spontaneously) represented in a canonical (ascending) order, and therefore connected to spatial position markers, which could provide a basis for the reciprocal SNARC effect. Hence, on these assumptions, the WM account does not readily appear to predict reciprocal SNARC effects, and therefore seems to face problems when attempting to explain the reciprocal SNARC effect observed in our Experiment 3.

## Conclusion

The results of the present study demonstrate a dissociation between regular SNARC effects (in number–location tasks) and reciprocal SNARC effects (in location–number tasks) with vocal responses. While regular SNARC effects occurred in our experiments with different stimulus sets (digits, number words), reciprocal SNARC effects only occurred with location word stimuli and vocal number responses, but not with physical location stimuli and vocal number responses. These findings suggest that the associations between numerical and spatial information, which are responsible for regular and reciprocal SNARC effects, are completely bidirectional, and create symmetrical S-R compatibility effects, only for particular combinations of stimulus and response sets. For other combinations of stimulus and response sets, the associations are unidirectional (i.e., number ➔ space) and allow for regular SNARC effects only. The present results complement our previous findings of SNARC effects in the absence of reciprocal SNARC effects with manual responses. Together, the findings have implications for existing theoretical accounts of the SNARC effect which need to explain that stimulus mode affects the emergence of reciprocal but not regular SNARC effects.

## Figures and Tables

**Fig. 8:**
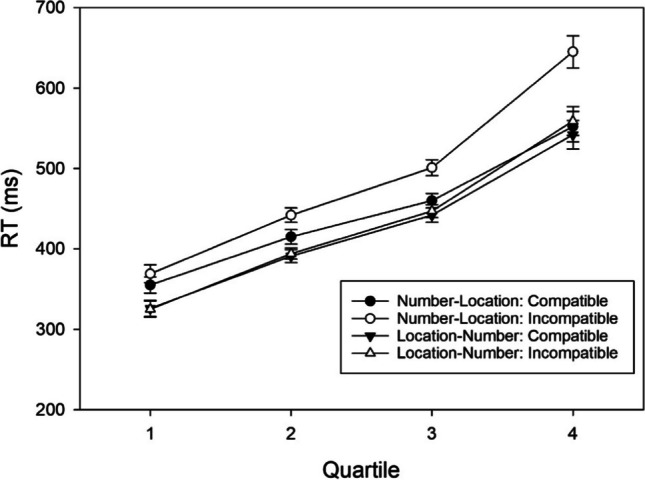
Mean RTs as a function of RT quartile, task, and S-R mapping observed in Experiment 1. Error bars reflect 95% confidence intervals for within-subjects designs (Cousineau, 2017)

**Fig. 9:**
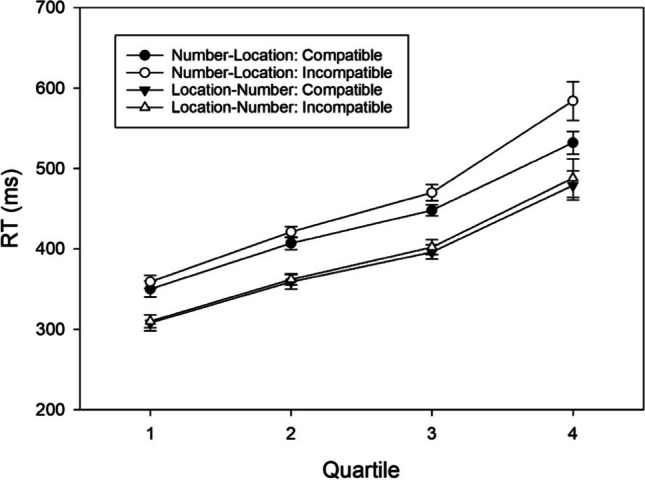
Mean RTs as a function of RT quartile, task, and S-R mapping observed in Experiment 2. Error bars reflect 95% confidence intervals for within-subjects designs (Cousineau, 2017)

**Fig. 10:**
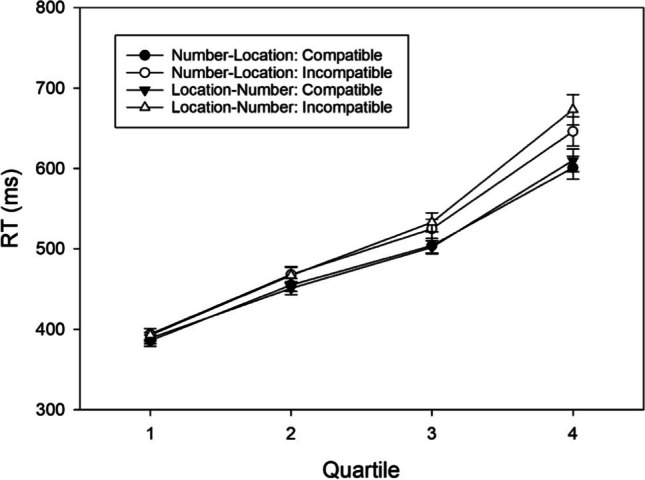
Mean RTs as a function of RT quartile, task, and S-R mapping observed in Experiment 3. Error bars reflect 95% confidence intervals for within-subjects designs (Cousineau, 2017)

## Data Availability

The data sets for the experiments reported here are available at the Mendeley repository [10.17632/dvpwx4634t.1]. All other materials can be obtained from both authors upon request.

## Appendix

### Distributional analysis for RTs in Experiment 1

For analyzing the time course of the mapping effects, we employed Ratcliff’s method of Vincentizing (Ratcliff, 1979). For each participant and condition, we rank-ordered RTs and subsequently divided this set of rank-ordered RTs into four quartiles. Means were computed for each quartile and then subjected to a three-factorial ANOVA with Task (number–location task, location–number task), Mapping (compatible, incompatible) and Quartile (1–4) as within-subject variables. Figure 8 illustrates the corresponding means. In this analysis, the interactions between Quartile and the other variables were of specific interest.

The two-way interactions Quartile × Task and Quartile × Mapping were both significant. The significant Quartile × Task interaction, *F*(3, 174) = 2.707, *MSE* = 742.524, *p* = .047, η_p_^2^ = .045, indicated a slightly larger increase in RTs in the number–location task compared with the location–number task for the latter quartiles. The significant Quartile × Mapping interaction, *F*(3, 174) = 52.244, *MSE* = 494.784, *p* < .001, η_p_^2^ = .474, indicated an increasing mapping effect with increasing RTs. Most importantly, however, the three-way interaction, *F*(3, 174) = 15.195, *MSE* = 713.305, *p* < .001, η_p_^2^ = .208, was also significant, revealing that the mapping effect had different time courses in the two tasks.

To determine the source of the significant three-way interaction, we conducted separate two-factorial ANOVAs, with Mapping and Quartile as within-subject variables for the two tasks. In case of significant interactions, we used Tukey’s post hoc tests due to the nondirectional hypothesis in the location–number task and to prevent alpha inflation. In the number–location task, the main effect of Mapping, *F*(1, 58) = 40.704, *MSE* = 5,632.726, *p* < .001, η_p_^2^ = .412, was significant. Importantly, the Mapping × Quartile interaction, *F*(3, 174) = 43.547, *MSE* = 805.604, *p* < .001, η_p_^2^ = .429, was also significant, reflecting that the regular SNARC effect increased with increasing RTs. While the mapping effect was 15 ms in the first quartile, it increased to 28 ms in the second quartile, 41 ms in the third quartile and 93 ms in the fourth quartile. Post hoc tests revealed that the regular SNARC effect was nonsignificant in the first quartile, *t*(58) = 2.918, *p*_*Tukey*_ = .087, but significant for all three larger quartiles, all *t*s ≥ 5.128, all *p*s_*Tukey*_ < .001. This finding is consistent with the established time course pattern of SNARC effects (e.g., Gevers et al., 2006; Mapelli et al., 2003).

In the location–number task, the main effect of Mapping, *F*(1, 58) = 2.139, *MSE* = 2,012.483, *p* = .149, η_p_^2^ = .036, did not reach significance, indicating the absence of a reciprocal SNARC effect. However, the Mapping × Quartile interaction, *F*(3, 174) = 3.991, *MSE* = 402.485, *p* = .009, η_p_^2^ = .064, was significant, revealing an increase in the RT difference between the compatible and incompatible condition with increasing RTs. This difference increased from 0 ms in the first quartile to 2 ms in the second quartile, 6 ms in the third quartile and 17 ms in the fourth quartile. Post hoc tests revealed that the reciprocal SNARC effect was nonsignificant in all four quartiles, all *t*s ≤ 2.004, all *p*s_*Tukey*_ ≥ .488.^8^

After excluding outliers according to the Tukey (1977) criterion, the interaction effect (Mapping × Quartile) in the location–number task became nonsignificant, *F*(3, 150) = 1.981, *MSE* = 399.097, *p* = .119, η_p_^2^ = .038, revealing that the increase in the reciprocal SNARC effect with increasing RTs vanished when outliers were excluded.

### Distributional analysis of RTs in Experiment 2

We conducted a distributional analysis in the form of a three-factorial ANOVA, with Task (number–location task, location–number task), Mapping (compatible, incompatible) and Quartile (1–4) as within-subject variables for Experiment 2. The distributional analysis was not pre-registered and must thus be considered an exploratory analysis. Figure 9 illustrates the corresponding means.

The two-way interactions Quartile × Task and Quartile × Mapping were both significant. The Quartile × Task interaction, *F*(3, 171) = 10.908, *MSE* = 811.185, *p* < .001, η_p_^2^ = .161, revealed a significantly larger increase in RTs in the number–location task compared with the location–number task for higher quartiles. The significant Quartile × Mapping interaction, *F*(3, 171) = 9.996, *MSE* = 728.997, *p* < .001, η_p_^2^ = .149, reflected an increasing mapping effect with increasing RTs. Yet, most importantly, the three-way interaction, *F*(3, 171) = 8.433, *MSE* = 456.066, *p* < .001, η_p_^2^ = .129, was also significant, indicating different time courses of the mapping effect in the two tasks.

To unravel the significant three-way interaction, we conducted two-factorial ANOVAs with Mapping and Quartile as within-subject factors for each of the two tasks. In the number–location task, the main effect of Mapping, *F*(1, 57) = 19.729, *MSE* = 3,452.889, *p* < .001, η_p_^2^ = .257, was significant. Moreover, the Mapping × Quartile interaction, *F*(3, 171) = 19.307, *MSE* = 560.859, *p* < .001, η_p_^2^ = .253, was significant, revealing that the regular SNARC effect increased with increasing RTs. Specifically, the mapping effect was 9 ms in the first quartile, 14 ms in the second quartile, 22 ms in the third quartile and 52 ms in the fourth quartile. Post hoc tests between the compatible and incompatible mapping condition for each quartile revealed that the regular SNARC effect was nonsignificant in the first quartile, *t*(57) = 2.203, *p*_*Tukey*_ = .366, but significant in all three larger quartiles, all *t*s ≥ 3.176, all *p*s_*Tukey*_ ≤ .046. Again, this time course pattern is congruent with the one that has been empirically established for SNARC effects (e.g., Gevers et al., 2006; Mapelli et al., 2003).

In the location–number task, the main effect of Mapping, *F*(1, 57) = 0.634, *MSE* = 4,315.829, *p* = .429, η_p_^2^ = .011, was nonsignificant, reflecting the absence of a reciprocal SNARC effect. Moreover, the Mapping × Quartile interaction, *F*(3, 171) = 0.488, *MSE* = 624.205, *p* = .691, η_p_^2^ = .008, was also nonsignificant, indicating that no small reciprocal SNARC effect emerged at particular RT levels.

After excluding outliers according to the Tukey (1977) criterion, in the location–number task the effect size of the nonsignificant mapping effect, *F*(1, 53) = 0.002, *MSE* = 1435.527, *p* = .963, η_p_^2^ < .001, and the effect size of the nonsignificant interaction effect (Mapping × Quartile),* F*(3, 159) = 0.017, *MSE* = 481.381, *p* = .997, η_p_^2^ < .001, further decreased towards zero.

### Distributional analysis of RTs in Experiment 3

We conducted a distributional analysis in the form of a three-factorial ANOVA, with Task (number–location task, location–number task), Mapping (compatible, incompatible) and Quartile (1–4) as within-subject variables for Experiment 3. Figure 10 illustrates the corresponding means. The two-way interactions Quartile × Task and Quartile × Mapping were both significant. The Quartile × Task interaction, *F*(3, 147) = 3.874, *MSE* = 957.503, *p* = .011, η_p_^2^ = .073, revealed a significantly larger increase in RTs in the location–number task compared with the number–location task for higher quartiles. The significant Quartile × Mapping interaction, *F*(3, 147) = 19.687, *MSE* = 1,128.355, *p* < .001, η_p_^2^ = .287, reflected an increasing mapping effect with increasing RTs. The three-way interaction, *F*(3, 147) = 1.499, *MSE* = 663.751, *p* = .217, η_p_^2^ = .030, was nonsignificant, indicating similar time courses of the mapping effect in the two tasks.

After excluding outliers according to the Tukey (1977) criterion, the three-way interaction, *F*(3, 135) = 3.213, *MSE* = 610.619, *p* = .025, η_p_^2^ = .067, became significant, revealing a greater increase of the mapping effect with increasing RTs for the location–number task than for the number–location task.

### Comparison of mapping effects between experiments

We conducted additional analyses to investigate how the changes between experiments affected the regular and the reciprocal SNARC effects, respectively. For each task, we therefore conducted a separate two-factorial ANOVA, with Mapping (compatible vs. incompatible mapping) as a within-subjects factor, Experiment (Exp. 1 vs. Exp. 2 vs. Exp. 3) as a between-subjects factor, and RTs from trials with correct responses as a dependent variable. For the number–location task, the main effect of Experiment was significant, *F*(2, 164) = 8.422, *MSE* = 8,467.036, *p* < .001, η_p_^2^ = .093, indicating differences in RTs between experiments. Post hoc test revealed significantly slower responses in Experiment 3 than in Experiment 2, *t*(164) = 4.103, *p*_*Tukey*_ < .001. The Mapping × Experiment interaction was also significant, *F*(2, 164) = 3.206, *MSE* = 1,303.311, *p* = .043, η_p_^2^ = .038, revealing differences in the regular SNARC effect between experiments. To unravel the interaction, we compared the mapping effect within each pair of experiments. The Mapping × Experiment interaction was significant for Experiments 1 and 2, *F*(1, 115) = 5.185, *MSE* = 1,127.643, *p* = .025, η_p_^2^ = .043, and for Experiments 1 and 3, *F*(1, 107) = 4.208, *MSE* = 1,540.093, *p* = .043, η_p_^2^ = .038, revealing a larger mapping effect in Experiment 1 compared with Experiment 2 and 3, respectively. For Experiments 2 and 3, *F*(1, 106) = 0.038, *MSE* = 1,254.878, *p* = .845, η^2^_p_ = .000, the interaction was not significant, indicating similar mapping effects in Experiment 2 and Experiment 3.

For the location–number task, the main effect of Experiment was significant, *F*(2, 164) = 48.306, *MSE* = 7,413.406, *p* < .001, η_p_^2^ = .371, indicating differences in RTs between experiments. Post hoc test revealed significantly slower responses in Experiment 1 than in Experiment 2, *t*(164) = 3.727, *p*_*Tukey*_ < .001, and significantly slower responses in Experiment 3 than in Experiment 1, *t*(164) = 6.217, *p*_*Tukey*_ < .001. The Mapping × Experiment interaction was also significant, *F*(2, 164) = 3.682, *MSE* = 1,294.681, *p* = .027, η_p_^2^ = .043, revealing differences in the reciprocal SNARC effect between experiments. The Mapping × Experiment interaction was significant for Experiments 1 and 3, *F*(1, 107) = 5.025, *MSE* = 1,418.181, *p* = .027, η_p_^2^ = .045, and for Experiments 2 and 3, *F*(1, 106) = 4.342, *MSE* = 1,736.474, *p* = .040, η_p_^2^ = .039, revealing a larger mapping effect in Experiment 3 compared with Experiment 1 and 2, respectively. For Experiments 1 and 2, *F*(1, 115) = 0.011, *MSE* = 772.554, *p* = .918, η_p_^2^ = .000, the interaction was not significant, indicating similar mapping effects in Experiment 1 and Experiment 2.

## Footnotes

1 Even though we use the term *regular SNARC effect* to denote the compatibility effect between numerical stimuli and spatial responses as employed in a typical SNARC task, we do not mean to characterize the effect in the opposite direction as *irregular*.

2 We use the term *reciprocal SNARC effect* to denote a compatibility effect between spatial stimuli and numerical responses.

3 Participants responded with the German words for “left”, “right”, “one”, “two” and “nine” in our experiments, but we are using the English words in the text for clarity.

4 Please note that, even though referred to within the discussion sections of the main manuscript, the distributional analyses of Exp. 1–3 (before and after the exclusion of outliers) are reported in the Appendix.

5 The experiment was pre-registered at OSF (osf.io/7kt4y).

6 In fact, the recordings of several vocal responses from these participants had poor quality, making it impossible to determine the accuracy of these responses. Note that we removed these participants (numbers 35, 36, 37) from the dataset.

7 Please note that, even though referred to within the discussion sections of the main manuscript, the comparison of mapping effects between experiments is reported in the Appendix.

## Code availability

The experiments’ programs (E-Prime 3.0) and the R code can be obtained from both authors upon request.
